# THE IMPACT OF THE COVID-19 PANDEMIC ON DEMENTIA CARE: A MULTISTAKEHOLDER PERSPECTIVE

**DOI:** 10.1093/geroni/igac059.475

**Published:** 2022-12-20

**Authors:** Sara Masoud, Carole White, Ashlie Glassner, Shanae Rhodes, Mayra Mendoza, Kylie Meyer

**Affiliations:** UT Health San Antonio, San Antonio, Texas, United States; UT Health San Antonio, San Antonio, Texas, United States; UT Health San Antonio, San Antonio, Texas, United States; UT Health San Antonio, San Antonio, Texas, United States; UT Health San Antonio, San Antonio, Texas, United States; Case Western Reserve University, Cleveland, Ohio, United States

## Abstract

Persons living with dementia (PLWD) and family caregivers are particularly vulnerable to the effects of the COVID-19 pandemic. A multi-methods study was conducted to describe the impact of the pandemic on dementia care from the perspectives of stakeholders, including PLWD, family caregivers, and health and social care professionals (HCPs).The study was conducted using a community engaged approach. Cross-sectional surveys were conducted with PLWD (n=27), family caregivers (n=161), and HCPs (n=77), followed by focus groups and interviews with a sub-sample of survey participants (n=55). Participants reported declines in health and quality of life for PLWD and family caregivers. Participants experienced delayed or cancelled dementia care attributed to the pandemic. Most reported telehealth and tele-support were effective alternative models to care. The pandemic impacted the quality and accessibility of dementia care. Results highlight opportunities to improve quality of care through addressing inequities and identifying approaches to address isolation and virtual care.

